# Effects of Local Administration of Iron Oxide Nanoparticles in the Prefrontal Cortex, Striatum, and Hippocampus of Rats

**DOI:** 10.1007/s12640-021-00432-z

**Published:** 2021-10-27

**Authors:** Ellen Irrsack, Julia Schuller, Charlotte Petters, Wiebke Willmann, Ralf Dringen, Michael Koch

**Affiliations:** 1 Department of Neuropharmacology, Centre for Cognitive Sciences, University of Bremen, PO Box 330440, 28334 Bremen, Germany; 2 Centre for Biomolecular Interactions Bremen (CBIB), and Centre for Environmental Research and Sustainable Technology, University of Bremen, PO Box 330440, 28334 Bremen, Germany

**Keywords:** Iron oxide nanoparticles, Neurodegeneration, Glial cells, Neurons, Immunocytochemistry, Neurotoxicity

## Abstract

Iron oxide nanoparticles (IONPs) are used for diverse medical approaches, although the potential health risks, for example adverse effects on brain functions, are not fully clarified. Several in vitro studies demonstrated that the different types of brain cells are able to accumulate IONPs and reported a toxic potential for IONPs, at least for microglia. However, little information is available for the in vivo effects of direct application of IONPs into the brain over time. Therefore, we examined the cellular responses and the distribution of iron in the rat brain at different time points after local infusion of IONPs into selected brain areas. Dispersed IONPs or an equivalent amount of low molecular weight iron complex ferric ammonium citrate or vehicle were infused into the medial prefrontal cortex (mPFC), the caudate putamen (CPu), or the dorsal hippocampus (dHip). Rats were sacrificed 1 day, 1 week, or 4 weeks post-infusion and brain sections were histologically examined for treatment effects on astrocytes, microglia, and neurons. Glial scar formation was observed in the mPFC and CPu 1 week post-infusion independent of the substance and probably resulted from the infusion procedure. Compared to vehicle, IONPs did not cause any obvious additional adverse effects and no additional tissue damage, while the infusion of ferric ammonium citrate enhanced neurodegeneration in the mPFC. Results of iron staining indicate that IONPs were mainly accumulated in microglia. Our results demonstrate that local infusions of IONPs in selected brain areas do not cause any additional adverse effects or neurodegeneration compared to vehicle.

## Introduction

During the last decade, iron oxide nanoparticles (IONPs) became increasingly useful for biomedical applications because of their biocompatibility, magnetic properties, and stability (Xie et al. 2018). Moreover, IONPs are used as contrast agents in magnetic resonance imaging (MRI), for cell tracking and for the treatment of brain tumors via magnetic hyperthermia (Bulte and Kraitchman 2004; Maier-Hauff et al. 2011; Petters et al. 2014; White et al. 2015; Marekova et al. 2020; Lorkowski et al. 2021). However, the risk of IONP treatment for human health especially for brain function is still not fully clarified. IONPs are able to enter the brain e.g. via the olfactory nerve after inhalation, cross the blood brain barrier after systemic injections or digestion and through direct intracranial injections (Muldoon et al. 1995; Mykhaylyk et al. 2001; Jain et al. 2008; Kwon et al. 2008; Wang et al. 2010, 2011a, b).

Several studies reported a toxic potential of IONPs on brain tissue caused by formation of reactive oxygen species (ROS) via metal-dependent Fenton reaction (Voinov et al. 2011; Yarjanli et al. 2017; Mai and Hilt 2019; Vakili-Ghartavol et al. 2020). Cell culture studies showed that astrocytes and neurons accumulate substantial amounts of IONPs and that such treatments do not compromise cell-viability (Geppert et al. 2012; Pinkernelle et al. 2012; Petters and Dringen 2014). In contrast, the strong accumulation of IONPs by cultured microglia severely impairs the cellular viability (Pickard and Chari 2010; Luther et al. 2013; Petters et al. 2016). Intranasal instillation of IONPs has been reported to increase the number of activated microglia in olfactory bulb, striatum and hippocampus (Wang et al. 2011a, b; Wu et al. 2013) and to cause oxidative stress in hippocampus, olfactory bulb and cerebellum (Wang et al. 2009) leading to neurodegeneration in the CA3 region of the hippocampus (Wang et al. 2007). Also, after systemic application, IONP accumulation, elevated NO levels, increased acetylcholinesterase activity, lactate dehydrogenase leakage, and demyelination were detected (Dhakshinamoorthy et al. 2017). These studies indicate the importance of further investigating the toxic potential of IONPs on brain cells in vivo as well as the ability of the brain to recover from IONP-induced neurodegeneration.

Here, we present data from a study investigating the effects of a local infusion of dimercaptosuccinic acid (DMSA)-coated IONPs in the medial prefrontal cortex (mPFC), the caudate putamen (CPu), or the dorsal hippocampus (dHip). Saline or ferric ammonium citrate was applied to control for damage inflicted by the injection procedure or by a low molecular weight iron compound. Rats were sacrificed 1 day (1d), 1 week (1w), or 4 weeks (4w) post-infusion, and brain sections were processed histochemically and immunohistochemically to assess the iron distribution, viability of neurons and activation of glial cells.

## Materials and Methods

### Synthesis and Characterization of Iron Oxide Nanoparticles

IONPs were synthesized by chemical co-precipitation of ferric and ferrous iron as previously described (Geppert et al. 2009). The IONPs were subsequently coated with dimercaptosuccinic acid (DMSA) that had been labeled with the fluorochrome BODIPY® FL C_1_-IA [*N-*(4,4-difluoro-5,7-dimethyl-4-bora-3a,4a-diaza-s-indacene-3-yl)methyl) iodoacetamide] as previously described in detail (Rastedt et al. 2017). The hydrodynamic diameter and the ζ-potential of the IONPs (1 mM total iron content) dispersed in 0.9% sterile saline was determined by dynamic and electrophoretic light scattering in a Beckman Coulter (Krefeld, Germany) Delsa™ Nano C Particle analyzer as reported (Rastedt et al. 2017). The hydrodynamic diameter of the IONPs dispersed in saline was 52.9 ± 7.4 nm and the ζ-potential of the IONPs was − 35.4 ± 0.5 mV (mean values ± standard deviation of three independent syntheses of IONPs).

The fluorescent IONPs were injected into rat brains as described below. However, all attempts failed to localize fluorescent IONPs in brain tissue and to co-localize IONPs with cell-type specific markers in vivo (data not shown) probably because the fluorescent dye in the coat of the IONPs is destroyed by the fixation and the staining conditions applied, and/or due to bleaching of the dye during handling of the sections and/or separation from the IONPs after uptake into cells, as recently demonstrated for cultured astrocytes (Willmann and Dringen 2018). Hence, fluorescence labelling of IONPs cannot be presented as intended.

### Animals

A total of 53 adult male *Wistar rats* (purchased from Charles River, Sulingen, Germany) were housed in standard Macrolon type IV cages under controlled ambient conditions (12 h light/dark cycle, lights on at 7:00 a.m., 45–55% humidity, 21–22 °C). They received rodent lab chow (12 g per rat per day) and had access to water ad libitum. Body weight of the animals was monitored throughout the study. The experiments were performed in accordance with the National Institutes of Health ethical guidelines for the care and use of laboratory animals for experiments and were approved by the local animal care committee (Senatorische Behörde, Bremen, Germany).

### Intracerebral Substance Administration

Substances administered intracerebrally were either sterile saline (0.9% NaCl) as vehicle (VEH), 1 mM ferric ammonium citrate (FAC; Roth, Karlsruhe, Germany) dissolved in sterile saline as ferric iron control (Bishop and Robinson 2001) or 1 mM iron as IONPs dispersed in sterile saline. The iron-containing solutions were prepared freshly before use and administered in a pseudorandomized order.

For intracerebral infusion into the mPFC, CPu, and dHip, rats were anesthetized with 60 mg/kg ketamine and 0.5 mg/kg medetomidine (CP-Pharma, Burgdorf, Germany) intraperitoneally (i.p.) and fixed in a stereotactic frame. To support cardiac and respiratory functions, 0.1 mg/kg atropine was given subcutaneously (Braun, Melsungen, Germany). Three holes were drilled in the skull unilaterally at the target positions according to the rat brain atlas of Paxinos and Watson (1998). The coordinates of the injection sites were as follows: mPFC—rostrocaudal −2.7 mm, lateral ±0.8 mm, ventrodorsal +3.7 mm; CPu—rostrocaudal −1.2 mm, lateral ±2.0 mm, ventrodorsal +5.0 mm; dHip—rostrocaudal +3.8 mm, lateral ±2.0 mm, ventrodorsal +3.4 mm. Stainless 30 gauge injection cannulas connected to microliter syringes (SGE Scientific Glass Engineering, Darmstadt, Germany) via polyethylene tubes were inserted and 0.5 µL of IONPs in saline, FAC in saline or VEH were injected at a rate of 0.2 µL/min. The cannula remained in the brain for additional 2.5 min to avoid substance reflux and to allow diffusion into the parenchyma. Subsequently, the cannula was withdrawn, the drill holes were closed with bone wax (SMI, Steinerberg, Belgium), and the skin was sutured. No treatment-related alterations in bodyweight or obvious behavioural changes during the experiments were observed.

### Preparation of Brain Tissue

The rats were euthanized 1d (*n* = 15), 1w (*n* = 18), or 4w post-surgery (*n* = 20) by an overdose of pentobarbital (200 mg/kg, i.p., Sigma-Aldrich, Steinheim, Germany) and transcardially perfused with 250 mL of ice-cold phosphate-buffered saline (PBS; 2.851% Na_2_HPO_4_·2H_2_O, 0.552% NaH_2_PO_4_·H_2_O and 0.9% NaCl in *aqua dest*., pH 7.4) followed by 250 mL of ice-cold 4% paraformaldehyde (PFA; Serva Electrophoresis, Heidelberg, Germany) in 0.1 M sodium phosphate buffer (PB) pH 7.4. The brains were post-fixed in 4% PFA in 0.1 M PB for 24 h, followed by cryoprotection in 30% sucrose solution (in 0.1 M PB) for 72 h. Six series of coronal brain section (40 µm) were cut on a cryostat (Jung CM 3000, Leica Instrument, Nussloch, Germany) and collected in PBS.

### Nissl Staining

For the detection of injection sites, brain sections were Nissl-stained with thionine by a standard method. Sections were mounted on gelatinized glass slides, dried, rehydrated by a descending alcohol row, stained in thionine, dehydrated via an ascending alcohol row and coverslipped with Entellan® (Merck, Darmstadt, Germany).

### Detection of Iron

For iron-detection in the brain tissue, free floating brain sections were processed at room temperature (RT) by a modified Perls’ staining protocol (Moos and Møllgård 1993). Briefly, free-floating sections were incubated for 30 min in 5% potassium ferrocyanide in 0.1 M PB and for further 30 min in 2% HCl + 5% potassium ferrocyanide. Subsequently, sections were rinsed twice for 10 min in PBS. Afterward, the sections were pre-incubated for 15 min in Tris-buffered saline (TBS; 1.32% tris(hydroxymethyl)aminomethane in PBS; 0.14% NaH_2_PO_4_·H_2_O, 0.02% KCl, 0.2% NaOH and 0.8% NaCl in *aqua dest*., pH 7.4). For intensification of the Perls’ staining, sections were transferred into a TBS solution containing 0.05% 3,3-diaminobenzidine tetrahydrochloride (DAB) and 0.07% imidazole. The reaction was started by adding 0.3% ammonium nickel sulfate and 0.01% H_2_O_2_ for 10 min. Finally, sections were rinsed in PBS, mounted onto gelatine-covered glass slides, air dried, dehydrated via graded alcohol solutions and coverslipped with Entellan.

### Detection of Degenerated Neurons

Fluorojade C (FJC) is a polyanionic fluorescein derivative which binds selectively to degenerated neurons that can be used to assess the time course of dying neurons (Schmued et al. 2005). Brain slices were mounted on Superfrost slides and dried overnight. The slides were first incubated at RT in 1% NaOH in 80% ethanol for 5 min, followed by 2-min incubation in 70% ethanol and 2 min in *aqua dest*. To suppress autofluorescence, the sections were incubated in 0.06% potassium permanganate solution for 10 min, followed by a tap water rinse for 1–2 min. Afterward, sections were incubated in 0.0002% FJC in 0.1% acetic acid for 15 min. Slides were washed three times each for 1 min in *aqua dest*. Slides were air dried, cleared in Roti Histol® and coverslipped with Entellan.

### Immunofluorescence

Immunohistochemical stainings were performed as described previously (Hayn and Koch 2015). Briefly, free-floating sections were rinsed three times in PBS for 10 min and incubated for 60 min at 4 °C in a blocking buffer consisting of PBS, 10% normal goat serum (Linaris, Wertheim-Bettingen, Germany), and 0.1% Triton-X-100 (Sigma-Aldrich, Steinheim, Germany). Afterward, sections were incubated for 72 h at 4 °C with rabbit anti-glial fibrillary acidic protein (GFAP, DAKO, Code no. Z0334, Denmark; 1:5000) for the detection of astrocytes, with anti-mouse nuclear neuronal marker (NeuN, EMD Millipore, MAB377, Germany) for the detection of neurons (1:1000) and with anti-Iba-1 (Wako, Germany; 1:2000) for the detection of microglia, respectively. After washing three times in PBS for 10 min, sections were blocked in a buffer containing 10% bovine serum albumin in PBS (PBSA) for 60 min at RT. Afterwards, sections were incubated for 48 h in biotinylated goat anti-rabbit antibody (GFAP and Iba-1) or in biotinylated goat anti mouse (NeuN) in 10% PBSA at 4 °C. Thereafter, sections were incubated in PBSA containing Alexa Fluor 594 streptavidin (1:2000, Sigma-Aldrich, Deisenhofen, Germany) for 24 h at RT. Finally, sections were mounted on gelatinized glass slides and underwent a further staining procedure with 0.5% Sudan Black (Acros Organics, Belgium) in 70% ethanol and two subsequent washing steps in PBS to suppress autofluorescence. The sections were coverslipped with fluorescence mounting medium (DAKO, Glostrup, Denmark).

### Image Analysis

Fluorescent and light microscopic images of tissue sections from injection sites were taken using a Zeiss Axioskop II microscope (Zeiss, Göttingen, Germany). For fluorescent images the appropriate band pass filter (excitation/emission peaks: Alexa Fluor 594 at 590 nm and 617 nm (red); BODIPY FL and FJC at 503 nm and 512 nm (green)) were used. Photomicrographs were taken by a digital camera RT slider spot connected to the image analysis software Metamorph 4.6 (Visitron Systems, Puchheim, Germany). The iron distribution at the injection sites in the different brain areas were evaluated qualitatively by observing the iron distribution in the tissue, in ramified and amoeboid brain macrophages (Bishop and Robinson 2001), but also in erythrocytes which were identified by their typical biconcave morphology. Quantification of the images was performed with the image processing software FIJI (Schindelin et al. 2012). The method for examination of the distribution of astrocytes at the infusion site was adapted from Hayn and Koch (2015). Briefly, to get binary images, 16-bit images were converted into 8-bit images and the contrast was enhanced about 0.3% followed by the application of an auto local threshold (method: median; radius: 80 pixel; correction value (c): −30). The astroglial density [%] underneath the needle tract was calculated for each region of interest (ROI) 450 × 450 µm (0.2 mm^2^). Because of the heterogeneity of the microglial shape and diameter, Iba-1-positive (Iba-1 +) cells were counted within a ROI using the cell counter plug-in. Iba-1 + cells were expected in the direct surrounding of the infusion-site; therefore, a ROI of 0.2 mm^2^ was evaluated. Only bushy and amoeboid microglia cells were taken into account. To quantify the neuronal populations at the infusion sites in the mPFC and CPu, 16-bit grayscale images were converted into 8-bit binary images as mentioned above. “Clotted” neurons were separated by the watershed function. Neurons were counted by FIJI’s “analyse particle” function within a ROI of 450 µm × 450 µm (0.2 mm^2^) underneath the injection-site and for evaluation extrapolated to 1 mm^2^. Because it is impossible to separate neurons in the dHip and to hit exactly the same positions with the cannula in every rat, white pixels were counted underneath the needle tract in a ROI of 150 µm × 300 µm (0.045 mm^2^) and were directly compared with the untreated hemisphere of the same rat. FJC-positive cells, which showed the morphological profile of neurons, were counted using the cell counter plug-in at the injection sites. For each immunohistological analysis, one adequate ROI was set within the anatomically defined confines of the selected brain regions on the treated (as well as on the untreated, contralateral brain hemisphere for control). In all cases the observer was blind to treatment.

### Statistical Analysis

For statistical analysis, SigmaStat software (Version 3.5 for Windows) was used. To assess the extent of tissue damage by the infusion procedure, the VEH-treated brain hemisphere was compared with the untreated contralateral hemisphere. Normally distributed data was analyzed by a paired t-test (GFAP and NeuN) and in the case of nonnormally distributed data, a Wilcoxon signed rank test (Iba-1) was used. Data from GFAP-immunohistology was normally distributed after log-transformation, NeuN for mPFC and CPu was normally distributed. Both immunohistological approaches were analysed by a two-way analysis of variance (2-way ANOVA) with the factors time (1d, 1w, and 4w) and treatment (VEH, FAC, and IONP). For post hoc multiple comparisons Tukey’s *t*-test was conducted and the level of significance was *p* < 0.05. Data of Iba-1-immunohistochemistry was not normally distributed so that a Kruskal–Wallis one-way ANOVA on ranks was used with the factor time within a treatment group and the factor treatment group within a time point. Post hoc Dunn’s method for multiple comparisons was used, and level of significance was *p* < 0.05. The data of GFAP- and NeuN immunohistology are presented in bar graphs and data of Iba-1 evaluation are shown as box plots.

## Results

### Nissl Staining

**Fig. 1:**
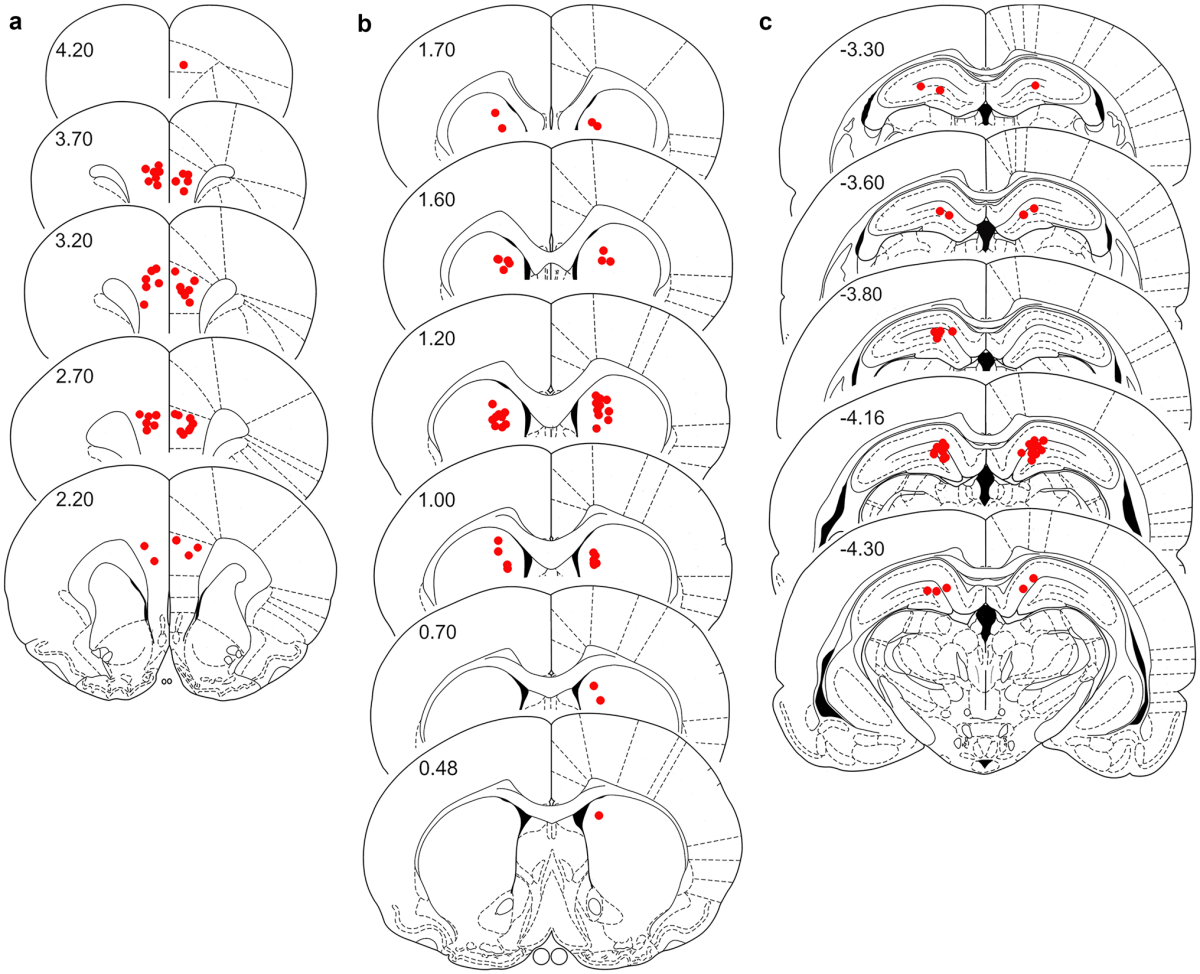
Location of the unilateral infusion-sites (red circles) in the medial prefrontal cortex (**a**), the caudate putamen (**b**), and the dorsal hippocampus (**c**). Rostral distance (mm) from Bregma is given by numbers. Schematic drawings are taken from the rat brain atlas of Paxinos and Watson (1998)

Microscopic analysis of Nissl-stained sections revealed that microinjections in 45 rats were located within the target areas (Fig. 1a–c). Eight rats were excluded from the evaluation because of misplaced cannulae (*n* = 2), respiratory failure during surgery (*n* = 3) or failure of the immunohistochemistry (*n* = 3). Brain areas which were mechanically damaged during preparation where also excluded from the analysis (*n* = 1). Therefore, three to five rats per treatment group per survival time were available for histological evaluation.

### Effects of Saline Infusions

It is known that a stab wound causes glial scar formation and an increased local immune response (Potter et al. 2012). Moreover, blood vessel rupture due to the infusion procedure or edema caused by fluid infusion was expected. Therefore, we first investigated the effects of saline infusions into the brain over the experimental period of up to 4w on the number of GFAP-positive cells as indicator of glial scar formation (Eng 1985; Sofroniew and Vinters 2010), the number of microglial cells, as indicator for microglial activation (Ito et al. 1998; Ahmed et al. 2007), as well as the number of neurons as indicator for a loss of neurons (Wolf et al. 1996).

**Fig. 2:**
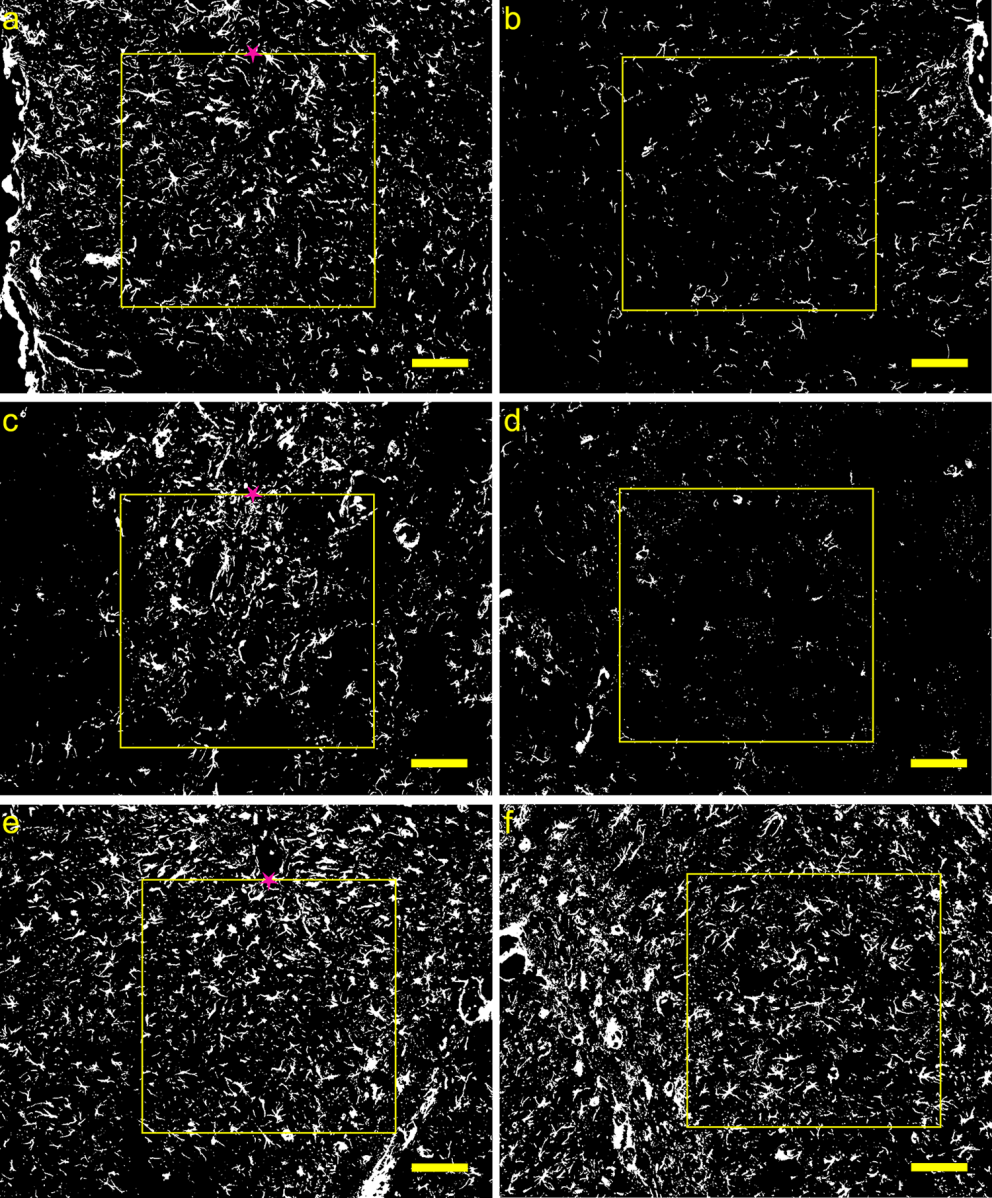
Representative binary images of immunohistological GFAP staining in the medial prefrontal cortex (**a**, **b**), caudate putamen (**c**, **d**), and the dorsal hippocampus (**e**, **f**) 1w after unilateral vehicle-infusion. Panels on the left side show brain hemispheres with infusion-sites marked by a pink star. Panels on the right side show the appropriate contralateral nontreated hemisphere of the same rat. The yellow squares delineate the region of interest (0.2 mm^2^). *Scale bar* = 100 µm

For the investigation of the VEH infusion, the untreated contralateral hemisphere served as reference. Immunohistochemical staining for GFAP demonstrated an increase in the density of astrocytes in the injected hemisphere compared to the nontreated hemisphere which was prominent 1w after injection of saline in all three brain regions investigated (Fig. 2). Quantitative analysis revealed no significant effects 1d post-surgery, while at least 1w post-surgery in all three regions a significant increase of astrocytic density was found (Table 1).

**Table 1:** Effects of saline infusions on the density of GFAP-positive astrocytes

Area	Time	*n*	Treated hemisphere GFAP density [%]	Untreated hemisphere GFAP density [%]	*p*
mPFC	1d	5	2.99 ± 0.77	1.98 ± 0.48	0.177
	1w	5	8.97 ± 0.49	3.30 ± 0.64	0.003**
	4w	5	5.12 ± 1.22	1.90 ± 0.36	0.067
CPu	1d	5	1.63 ± 0.64	1.98 ± 0.48	0.501
	1w	5	6.41 ± 1.74	1.16 ± 0.18	0.005**
	4w	5	3.99 ± 0.71	1.37 ± 0.52	0.017*
dHip	1d	5	8.78 ± 1.27	7.44 ± 0.64	0.379
	1w	5	12.55 ± 2.49	6.29 ± 0.62	0.026*
	4w	5	7.87 ± 2.47	7.62 ± 1.52	0.946

GFAP density with a ROI of 0.2 mm^2^ in brain sections obtained from the different brain areas and hemispheres of rats that had been infused with saline. Data are shown as means ± SEM. Statistical analysis of the data obtained for the two hemispheres were done by paired *t*-test, and the level of significance is indicated by asterisks (*p < 0.05, **p < 0.01)

To test for microglial activation after saline injection into the brain, immunohistochemical staining was performed for Iba-1, which is upregulated during microglial activation (Ito et al. 1998; Ahmed et al. 2007; Yasuda et al. 2007). Although some increase in the number of microglial cells was observed for the injected hemisphere, the differences in Iba-1-positive cells between control and injected hemisphere did not reach the level of significance (Table 2).

**Table 2:** Effects of saline infusions on the number of Iba-1-positive cells

Area	time	n	Treated hemisphere Iba-1 + [n]	Untreated hemisphere Iba-1 + [n]	*p*
mPFC	1d	5	20 ± 3	12 ± 3	0.125
	1w	5	41 ± 12	15 ± 3	0.063
	4w	5	19 ± 5	16 ± 3	0.063
CPu	1d	5	13 ± 3	12 ± 1	0.625
	1w	5	21 ± 9	12 ± 1	0.063
	4w	5	19 ± 5	14 ± 1	0.125
dHip	1d	5	22 ± 3	16 ± 4	0.063
	1w	5	42 ± 6	15 ± 3	0.063
	4w	5	20 ± 2	14 ± 2	0.313

Pairwise comparison of microglial number within a frame of 0.2 mm^2^ between VEH-treatment and the contralateral hemisphere after 1d, 1w, and 4w in the mPFC, CPu, and dHip. Data are presented as median ± SEM. Level of significance was set on *p* < 0.05

Immunostaining for NeuN revealed for most of the regions and times investigated only insignificant differences in the number of neurons (Table 3). Only 4w after injection of saline into the mPFC, a significant loss of neurons in the injected hemisphere compared to the contralateral hemisphere was observed (Table 3). The number of NeuN + cells was insignificantly reduced 1d post-treatment in the mPFC (*p* = 0.055). No significant effects were found in the CPu and in the dHip.

**Table 3:** Effects of saline infusion on the number of NeuN-positive cells

Area	time	n	Treated hemisphere NeuN + [*n*]	Untreated hemisphere NeuN + [*n*]	*p*
mPFC	1d	5	372 ± 84	646 ± 73	0.055
	1w	3	655 ± 113	828 ± 37	0.238
	4w	5	507 ± 93	683 ± 101	0.01*
CPu	1d	5	768 ± 107	771 ± 97	0.979
	1w	3	830 ± 139	720 ± 149	0.249
	4w	5	750 ± 125	713 ± 105	0.699
dHip	1d	5	38% ± 2%	62% ± 1.56%	0.063
	1w	3	39% ± 2%	61% ± 1.95%	0.25
	4w	5	47% ± 4%	53% ± 4.27%	0.438

Pairwise comparison of neuronal number (NeuN-immunohistochemistry per mm^2^) between VEH-administration and its contralateral counterpart after 1d, 1w, and 4w in the mPFC and CPu. The histological images of dHip were converted to binary pictures and the percentages of white pixels representing NeuN + cells within a frame of 0.045 mm^2^ were determined. Number of neurons in the mPFC and CPu are shown as means ± SEM. The percentage of NeuN + cells in the dHip are expressed as median ± SEM. The significance of data for the neuronal number is indicated by asterisks. Level of significance was set on *p* < 0.05.

### Effects of Iron Infusions

#### Immunohistochemical Staining for Astrocytes

**Fig. 3:**
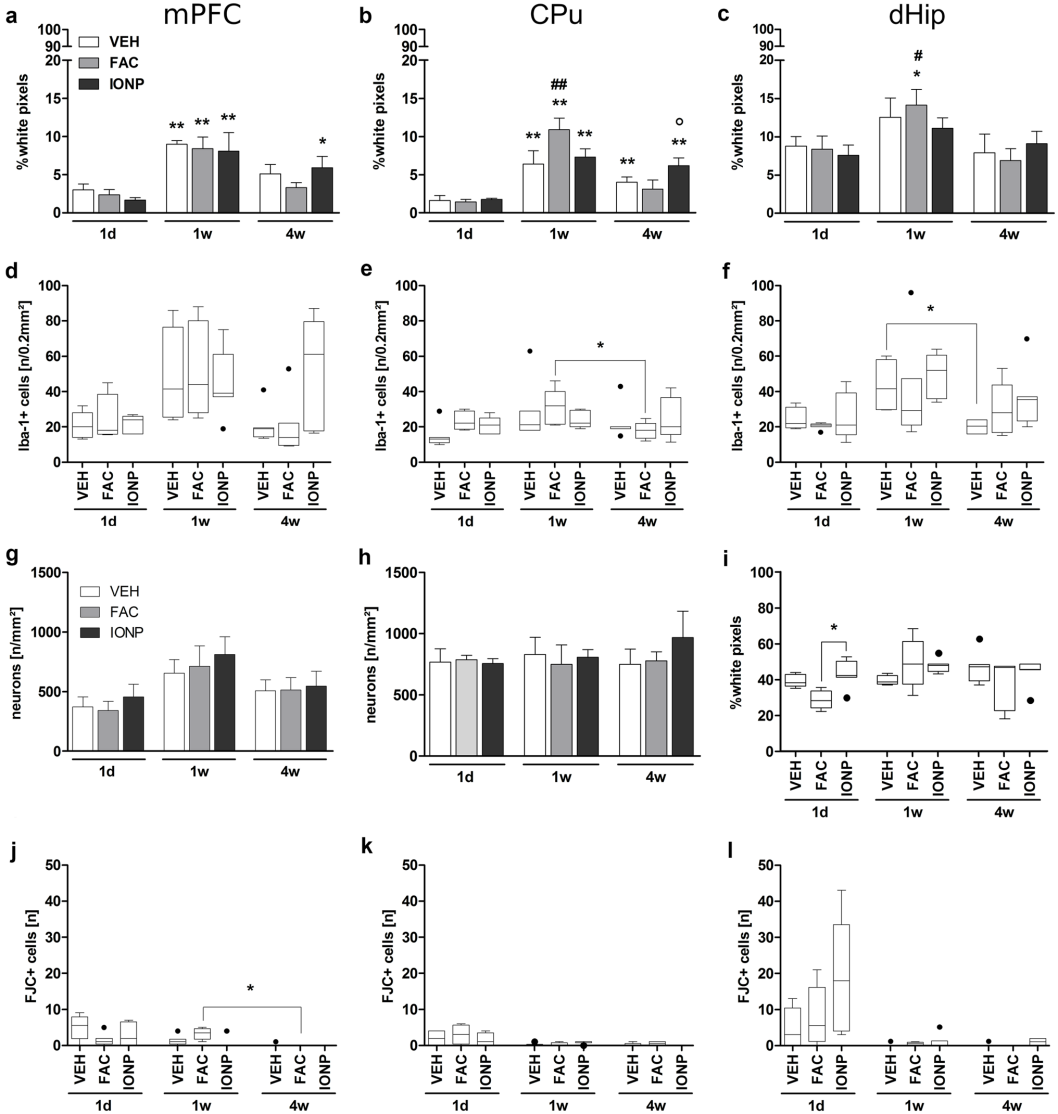
Quantitative histological analysis 1d, 1w, or 4w after infusion of vehicle (VEH), ferric ammonium citrate (FAC) or iron oxide nanoparticles (IONP) into the medial prefrontal cortex (mPFC; **a**, **d**, **g**, **j**), caudate putamen (CPu; **b**, **e**, **h**, **k**) or dorsal hippocampus (dHip; **c**, **f**, **i**, **l**). Bar graphs **a**–**c** showing the distribution of GFAP + cells at the injection site in a ROI of 0.2 mm^2^. Box plots **d**–**f** show the number of Iba1 + cells per 0.2 mm^2^ at the infusion site. Bar graphs g and h show the number of neurons per mm^2^ underneath the injection site and the box plot in panel **i** shows the density of neurons in the dHip. Panels **j**–**l** show the number of FJC + cells at the injection site. Data in the bar graphs are means ± SEM. Asterisks denote a significant difference at a certain time point compared to 1d within a substance-group (Tukey’s *t*-test; * *p* < 0.05; ** *p* < 0.01). Hashes denote a significant difference between a certain time point compared to 4w within a substance-group (Tukey’s *t*-Test; # *p* < 0.05; ## *p* < 0.01). White circle denotes a significant difference between a certain substance-group compared to the FAC-condition within the same time point (Tukey’s *t*-test; ○*p* < 0.05). Boxes of box plots represent the inter quartile range (IQR); the middle bar is the median value; whiskers mark the maxima and minima values within 1.5 × IQR. Values out of 1.5 × IQR are defined as outliers and are represented as black circles. The asterisks denote a significant difference (Dunn’s test; *p < 0.05). For immunohistological approaches the sample sizes was in almost all cases *n* = 5, aside from a few exceptions: rats received FAC 1w post-surgery in the dHip (*n* = 4) and the group which received VEH 1w post-surgery (*n* = 3). In the case of FJC staining *n* = 5 for following groups: 4w post-infusion of VEH in all areas; 1d post-infusion of IONPs in all areas; 1w post-infusion of IONPs in the mPFC. *n* = 4 for following groups: 1d and 1w post-infusion of VEH in all areas; 1d and 4w post-infusion of FAC and 1w post-infusion of FAC in the mPFC and CPu; 1w post-infusion of IONP in the CPu and dHip and 4w post-infusion of IONPs in all areas. For 1w post-infusion of FAC in the dHip *n* = 3

Treatment- or time-effects on the distribution of astrocytes after infusion of IONPs or FAC were assessed by GFAP-immunohistochemistry. Presence of GFAP demonstrated a glial scar formation 1w post-injection in the CPu and mPFC independent of the treatment. In the dHip, a glial scar was only observed 1w after FAC infusion. In the mPFC a significant main effect of time was found (F_2, 36_ = 19.774; *p* < 0.001). A higher astroglial density was found 1w compared to 1d after infusions of VEH (*p* = 0.009), FAC (*p* = 0.001), or IONPs (*p* = 0.002). After 4w, the astrocytic density was still increased in the IONP-group (*p* = 0.012; Fig. 3a).

In the CPu, strongest glial scar formation was found 1w post-treatment. Statistical analysis revealed a main effect of time (*F*_2, 36_ = 35.186; *p* < 0.001). Compared to 1d post-infusion of VEH, the astrocytic density was increased 1w (*p* < 0.001) and 4w post-infusion of VEH (*p* = 0.007). Similar effects were found following IONP infusion, where 1w post-treatment the astrocytic density was higher compared to 1d (*p* = 0.001). In contrast to 1d post-infusion of IONPs, the astrocytic density was still increased 4w post-infusion (*p* = 0.004). Further post hoc Tukey’s *t*-test showed that FAC infusion led to an increase of astrocytic density 1w compared to 1d post treatment (*p* < 0.001) as well as 4w compared to 1w post-treatment (*p* < 0.001). Moreover, 4w post-treatment with FAC, astrocytic density was lower compared to IONP infusions (*p* = 0.034; Fig. 3b).

In the dHip, a main effect was found for time (*F*_2, 35_ = 7.346; *p* = 0.002). Glial scar formation was prominent 1w post-infusion of FAC compared to 1d (*p* = 0.045) and 4w post-infusion (*p* = 0.01) (Fig. 3c).

#### Immunohistochemical Staining for Microglia

For the evaluation of the microglia response 1d, 1w, or 4w post-treatment with VEH, FAC, or IONP in the mPFC, CPu, and dHip, we used Iba-1 immunohistochemistry. Strongest Iba-1 staining was observed 1w post-treatment with FAC in the CPu and VEH in the dHip. A Kruskal–Wallis 1-way ANOVA on ranks revealed a significant effect for time after VEH-treatment in the mPFC (*p* = 0.041; *H* = 6.376). However, post hoc comparison via Dunn’s method did not reveal a statistical significance (Fig. 3d). In the CPu, statistical analysis revealed a significant effect of time (*p* = 0.038; *H* = 6.518). Post hoc comparisons indicate a higher number of microglia 1w compared to 4w post-infusion of FAC (*p* < 0.05; Fig. 3e). Statistical analysis revealed no significant treatment effects in the dHip. However, a significant main effect for time was found (*p* = 0.014; H = 8.571). Number of microglia was higher 1w compared to 4w after VEH-treatment (*p* < 0.05; Fig. 3f).

#### Immunohistochemical Staining for Neurons

In the dHip a decrease of neuronal density was observed 1d after FAC treatment compared to IONP. Moreover, in the mPFC, the number of neurons is reduced 1d compared to 1w post-treatment. A 2-way ANOVA revealed a significant main effect of time (*F*_2, 34_ = 5.748; *p* = 0.007) in the mPFC. Post hoc Tukey’s *t*-test revealed a significantly lower number of neurons 1d compared to 1w post-treatment (*p* = 0.005) independent of the substances applied (Fig. 3g). No effect was found in the CPu since the numbers of NeuN + cells were similar in all treatment-groups and did not change over time (Fig. 3h). A Kruskal Wallis 1-way ANOVA on ranks revealed a significant treatment effect after 1d (*H* = 7.58; *p* = 0.023). Post hoc Dunn’s test showed a lower neuronal density 1d post-treatment with FAC compared to IONPs (*p* < 0.05; Fig. 3i).

#### Detection of Degenerated Neurons

FJC staining was carried out for histological visualization of neuronal degeneration in the mPFC, CPu, and dHip after treatment with VEH, FAC, and IONPs 1d, 1w, and 4w post-surgery. Only a few dead neurons were found in all treated areas compared to the untreated contralateral side. After 4w, almost no FJC + cells were detected independent of area or treatment. Most neuronal degeneration was observed 1d post-infusion without any substance-specific effect (data not shown).

For mPFC the statistical analysis revealed a time effect (*p* = 0.03; *H* = 7.049) with significantly higher number of FJC + cells 1w compared to 4w after FAC-treatment (*p* ˂ 0.05; Fig. 3j). No significant effects were observed in the CPu or dHip (Fig. 3k, l).

#### Histochemical Staining of Brain Sections for Iron

**Fig. 4:**
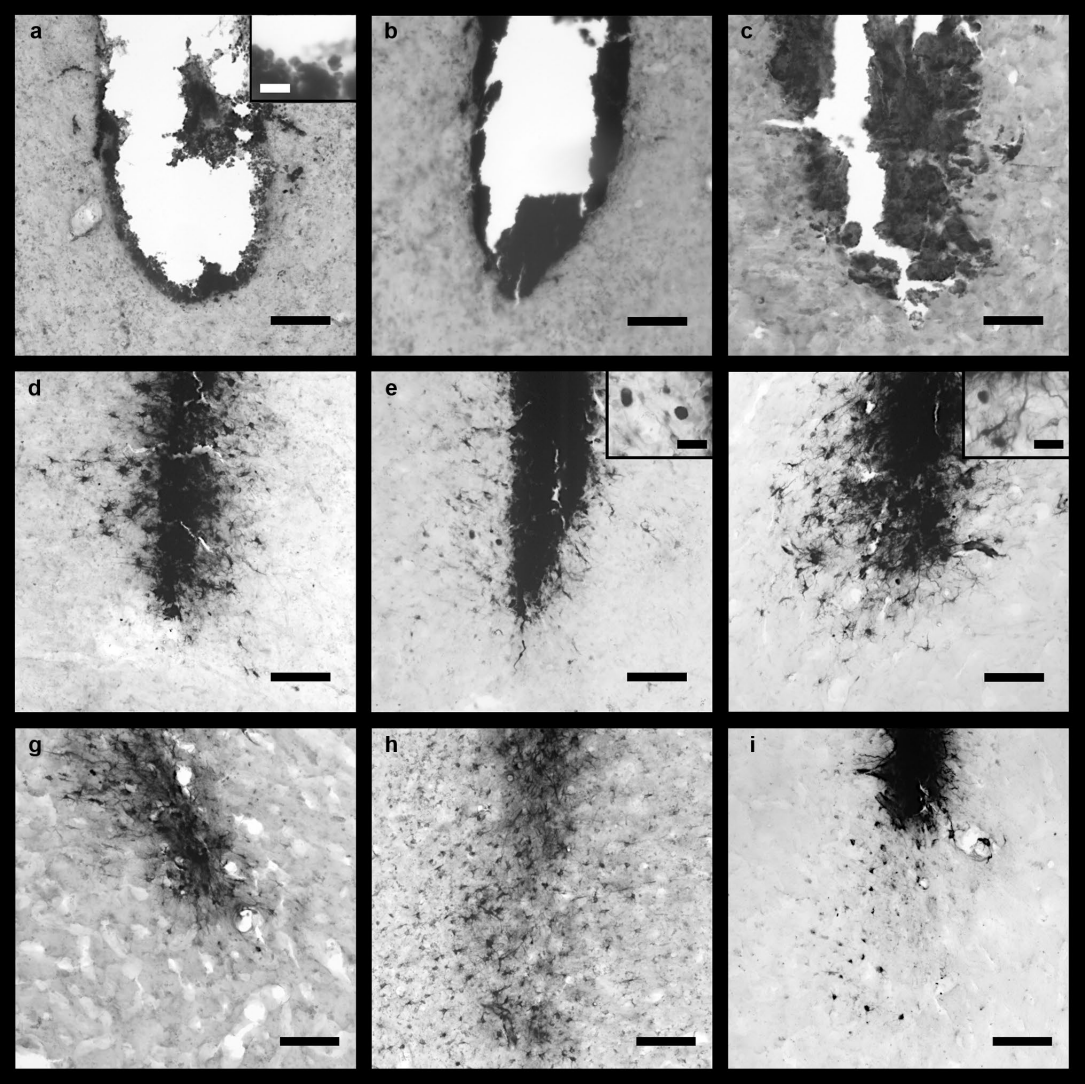
Representative microphotographs of rat brain sections of the mPFC stained with the modified Perl’s Prussian blue method and intensification with DAB after VEH (**a**, **d**, and **g**), FAC (**b**, **e**, and **h**), or IONP infusion (**c**, **f**, and **i**). One day post-infusion (**a**–**c**), mainly small iron + cells were observed. Because of their biconcave shape, they are identified as erythrocytes (see inset in **a**). Images **d**–**f** show injection sites 1w post-infusion. Many ramified iron + cells (inset in **f**) were observed in all treatment groups, but roundish iron + cells were observed mainly in the FAC and IONP groups (insets in **e** and **f**). Four weeks post-infusion, mainly ramified iron + cells were found (**g**–**i**). Widespread ramified cells were detected 4w after FAC (**h**) or IONP infusion (**i**) (*n* = 5 for each group). Scale bar = 100 µm; scale bar in the insets = 25 µm

Perls’ Prussian blue staining and DAB-intensification were used for the detection of iron in the brain tissue surrounding the injection site. Two types of iron loaded (iron +) cells were observed: ramified cells and amoeboid cells. Further iron + cells were observed directly at the needle tract and were identified as erythrocytes because of the biconcave shape (Fig. 4a, inset) and other blood cells which entered the stab wound via blood vessel rupture (e.g., Fig. 4c). In all cases, iron + cells were visible independent of treatment and time point. The iron staining was more intense in the IONP and FAC group in all brain areas; especially roundish amoeboid cells were mainly observed in these groups.

One day after treatment, the needle tract is clearly visible and erythrocytes as well as other blood cells were observed in the mPFC independent of the type of substance applied (Fig. 4a–c). One week post-infusion, the needle tract was still visible in all areas. Moreover, iron was also detected around the needle tract. All treatment-groups show ramified iron + cells around the infusion-site in the mPFC (Fig. 4d–f). However, iron + amoeboid cells were mainly observed after IONP and FAC treatment (Fig. 4e–f). Four weeks post-infusion, the infusion site is generally smaller, indicating wound healing independent of region and treatment. Many ramified iron + cells that had been migrated into the surrounding brain tissue were visible 4w after FAC treatment in the mPFC. In addition, the IONP group showed numerous iron + ramified cells, which were not only in the direct vicinity of the needle tract. However, 4w after VEH treatment many iron + ramified cells were visible, but mainly in close vicinity of the infusion site. Amoeboid cells were observed in all groups, especially in the FAC and IONP groups (Fig. 4g–i).

**Fig. 5:**
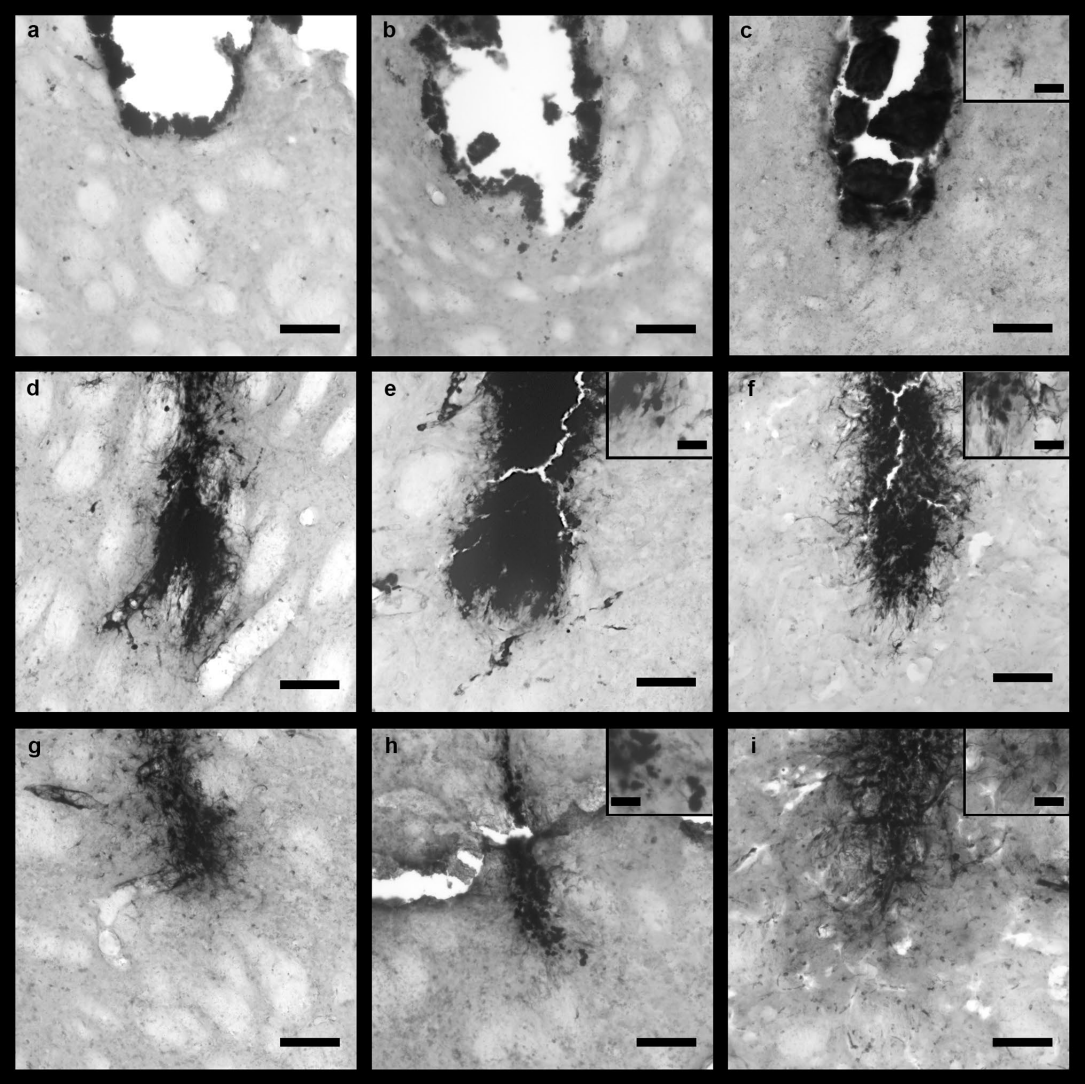
Representative microphotographs of rat brain sections of the CPu stained with the modified Perl’s Prussian blue method and intensification with DAB after VEH (**a**, **d**, and **g**), FAC (**b**, **e**, and **h**) or IONP infusion (**c**, **f**, and **i**). One day post-infusion (**a**–**c**) mainly small iron + cells were observed (presumably erythrocytes). However, two rats of the IONP-group showed iron + ramified cells (**c**). Images **d**–**f** show injection sites 1w post-infusion. Many ramified iron + cells (inset in **e** and **f**) were observed in all substance groups, but also roundish iron + cells were found in the FAC and IONP groups (insets in **e** and **f**). Four weeks post-infusion, mainly ramified iron + cells were observed (**g**–**i**). Widespread ramified cells were observed 4w after FAC (**h**) and IONP infusion (**i**), whereas after FAC infusion amoeboid cells were more abundant (**h**, inset). *n* = 5 for each group. Scale bar = 100 µm; scale bar in the insets = 25 µm

In addition to the abovementioned blood cells in the stab wound, few iron + ramified cells were found at the infusion site in the CPu in all treatment groups (Fig. 5c, inset). One week after FAC or IONP infusion into the CPu, many iron + ramified cells were detected in the brain parenchyma and also amoeboid cells were observed in these groups (Fig. 5e, f), while infusion of VEH resulted in less iron + cells (Fig. 5d). However, 4w post-infusion of VEH, many iron + ramified cells were observed in the surrounding of the needle tract (Fig. 5g). Nonetheless, iron + amoeboid cells were mainly found 4w post-infusion of FAC and IONPs (Fig. 5h, i).

**Fig. 6:**
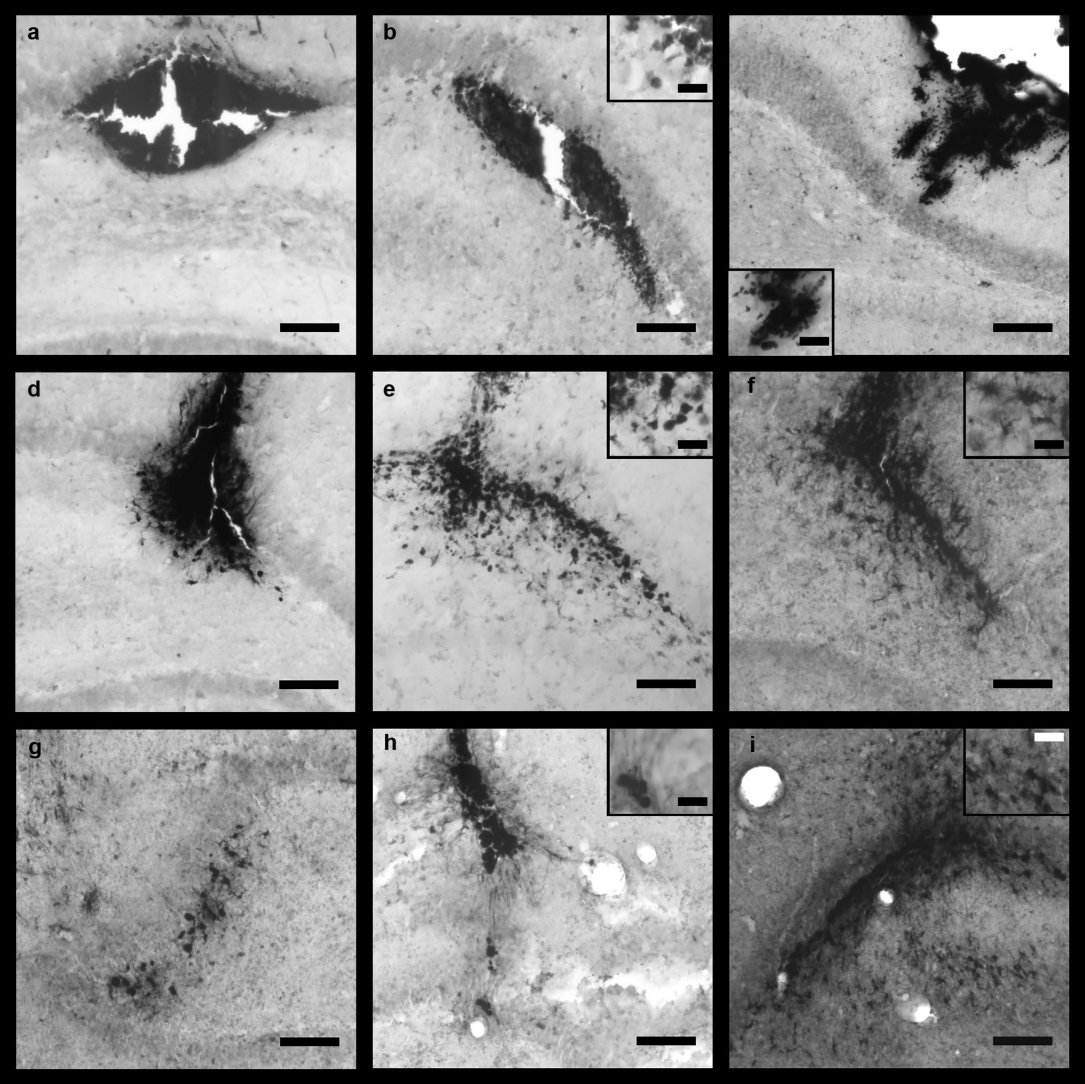
Representative microphotographs of brain sections of the dHip stained with the Perl’s Prussian blue method and intensification with DAB after VEH (**a**, **d**, **g**), FAC (**b**, **e**, **h**) or IONP infusion (**c**, **f**, **i**). One day post-infusion (**a**-**c**) mainly small iron + cells (presumably erythrocytes) were observed (inset **c**). However, few rats of the FAC group showed iron-positive amoeboid cells (**b**, inset). Images **d**–**f** show injection sites 1w post-infusion. Many ramified iron + cells (inset in **e** and **f**) were observed in all treatment groups, but also roundish iron + cells were observed mainly in the FAC-group (**e**, inset). Four weeks post-infusion, mainly ramified, but also amoeboid iron + cells were found in all groups (**g**–**i**). Widespread ramified cells were observed 4w after IONP infusion (**i**), whereas after FAC infusion amoeboid cells were often found (**h**, inset). *n* = 5 for each group, except *n* = 4 for 1w post-infusion of FAC. Scale bar = 100 µm; scale bar in the insets = 25 µm

In the dHip few Perls’ positive amoeboid cells were visible 1d after FAC, IONP, or VEH treatment (Fig. 6a–c). One week post-infusion of IONPs, high numbers of iron + ramified cells were found widespread in the dHip compared to VEH infusion. Likewise, in the FAC group iron + ramified cells were scarce, however, iron + amoeboid cells were observed to a greater extent than in the VEH and IONP groups (Fig. 6d–f). Four weeks post-infusion of VEH iron + ramified and very few amoeboid cells were found close to the injection site (Fig. 6g). Most amoeboid cells and many ramified cells were observed after 4w for the FAC and IONP groups (Fig. 6h–i).

## Discussion

Since the understanding of the fate of IONPs in the brain is still incomplete, the present study investigated the distribution of IONPs and possible tissue lesions at different time points after local infusion into three different brain areas compared with appropriate control infusions. Since cannula implantation and the infusion itself injures brain tissue, the VEH group was compared with the untreated contralateral brain hemisphere. The strongest tissue damage was observed 1w post-infusion as indicated by pronounced GFAP expression especially in the CPu, although the loss of neurons was moderate. Only in the mPFC neuronal loss was observed 4w post-treatment indicating a late response to mechanical injury by the infusion-cannula, edema, or extravasated hemoglobin (Dang et al. 2011; Zhou et al. 2017). Bishop and Robinson (2001) already demonstrated a high vulnerability of cortical neurons to mechanical impact as a result of a surgical intervention indicated by high number of FJC + cells around the injection tract. Likewise, in the present study, more FJC + cells were observed in the direct vicinity of the infusion tract in the mPFC supporting high vulnerability of cortical neurons to mechanical stress (data not shown). The comparison between VEH and the untreated hemisphere demonstrated the destructive effects of the infusion to the cortical tissue. Independent of the treatment, NeuN + positive cells decreased 1d post-infusion, which can be associated to the rupture of blood vessels and the ensuing inflammatory processes and to the fact that no migration of microglia and astrocytes to the infusion site took place so early. To protect surviving neurons from cell death by release of proinflammatory cytokines, cytotoxic proteases and ROS, astrocytes form a glial scar to limit the intrusion of blood-derived cells (e.g., leucocytes) from the injection site (Boghdadi et al. 2020). Furthermore, acute necrotic cell death due to CNS injuries results in secondary cell death by release of cytotoxic cell debris or an excess of glutamate into the extracellular space. The capability of microglia and astrocytes to phagocytize cell debris and the fact that astrocytes take up excessive glutamate from extracellular space prevents surviving brain cells from secondary cell death (Kawabori et al. 2015; Morizawa et al. 2017; Mahmoud et al. 2019; Boghdadi et al. 2020).

Perl’s Prussian blue staining showed lesions and mainly iron + biconcave erythrocytes supporting the assumption that hemin led to neuronal loss. Amoeboid iron + cells were abundant 1w post-infusion of FAC. Additional application of FAC led to an increased neurodegeneration after 1w, indicated by an increased number of FJC + cells. This might be related to the uptake of non-transferrin-bound iron (NTBI) which is reduced by membrane bound reductases to ferrous iron leading to ROS via the Fenton reaction (Bishop et al. 2011). With respect to neuronal viability, no adverse consequences were found after IONP treatment. This is consistent with data from cell culture experiments demonstrating that the IONPs used for the present study are internalized by cultured neurons and did even in millimolar concentration not acutely compromise the viability of these cells (Petters and Dringen 2015). As microglia at least in culture internalize IONPs more efficiently than neurons (Petters et al. 2016), it was hypothesized that also in the complex cellular system of the living brain, microglia may take up most of the injected IONPs before they could damage neurons. The qualitative evaluation of the Perls’ Prussian blue staining supports this assumption, since many iron + cells were identified as microglia due to their morphology. Moreover, 1w post-infusion, all treatment groups showed a strong glial scar formation accompanied by a slight and diffuse increase of microglia, and Perls’ method showed numerous iron-loaded ramified and amoeboid cells after IONP- and FAC-infusion, indicating uptake of iron. The density of GFAP + cells was increased in the IONP-group after 4w compared to 1d. The penetration of a foreign body leads to such a glial scar formation, which is usually accompanied by inflammatory cells like microglia (Sofroniew and Vinters 2010; Hayn and Koch 2015; Hayn et al. 2017).

Wu et al. (2013) reported that approximately 80% of IONPs remained in the striatum of rat brains 1w after intranasal instillation. Moreover, the striatum exhibited a greater vulnerability to oxidative stress compared to other brain regions. Furthermore, a previous study by Wang et al. (2011b) demonstrated enhanced presence of activated microglia 40 days after intranasal instillation. In our study, Perls’ staining showed a high presence of iron + cells in the CPu 1w after infusion of IONPs and FAC. Surprisingly, no impact on neurons was found in the CPu. Similar to the results in the mPFC, all interventions led to glial scar formation 1w post-surgery, no matter what substance was infused. A previous study showed that the immune reaction as well as the glial scar decrease over time after mechanical damage (Potter et al. 2012). In our study, the glial scar persisted 4w post-infusion of IONP and was more pronounced than after FAC infusion. One reason for this difference may be the coating material of IONPs that has a strong influence on the interaction between IONPs and biomolecules regarding the size, zeta potential, shape, and curvature (Nel et al. 2009; Salehipour et al. 2021). The type and coating of IONPs has already been reported to affect the distribution of IONPs after intracerebral infusion (Wang et al. 2019). The ligand coat stabilizes IONPs, while the iron core is responsible for magnetic properties (Laurent et al. 2008). The γ-Fe_2_O_3_ nanoparticles which were used in the study of Wang et al. (2011b) showed an altered crystal structure causing enhanced proliferation of microglial cells and higher release of NO and ROS by microglial cells, which may compromise neuronal viability. Furthermore, a previous study demonstrated a wide interstitial diffusion of Dextran-coated IONPs, whereas in the same study, gold-coated IONPs were only located at the needle tract probably due to the coating material. In both cases no strong elevation of astrocytes or activated microglia was detected (Wang et al. 2011a, b).

Previous in vivo studies demonstrated that intranasal instillation of IONPs led to enhanced iron levels in the rodent hippocampus, resulting in neurodegeneration in the CA3 area (Wang et al. 2007), formation of ROS (Wang et al. 2009), and microglial activation (Wang et al. 2011b). In our study, 1d after FAC and IONP infusion, iron + amoeboid cells were observed. The roundish morphology is characteristic for activated microglia (Bishop and Robinson 2001). Moreover, at all survival-times in the dHip most iron + amoeboid cells were observed after infusion of FAC and IONPs, suggesting that microglia take up FAC and IONPs. This is consistent with cell culture studies showing that cultured microglial cells take up IONPs more efficiently than other brain cells (Pinkernelle et al. 2012; Luther et al. 2013; Petters et al. 2016). In vivo, the number of microglia decreased 4w after VEH infusion, indicating that the surgical procedure itself does not cause a long-lasting microglial immune reaction in contrast to a treatment with iron. Moreover, in dHip the excess of NTBI in form of FAC causes the formation of a strong glial scar 1w post-infusion. In this scar, besides astrocytes also microglial cells are likely to play a neuroprotective role, since they efficiently accumulate extracellular NTBI (Bishop et al. 2011; Urrutia et al. 2013), which may lead to the increased number of iron + amoeboid cells observed after FAC-infusion for the time points investigated. Furthermore, the FAC-group showed a decrease in neuronal density in dHip 1d post-infusion which may be a consequence of iron accumulation. Previously, it was shown that rat primary hippocampal neurons are able to take up NTBI, which is reduced either by membrane-bound reductases by, e.g., Steap2 and following Zip8-mediated permeation of ferrous iron (Ji and Kosman 2015), promoting neurotoxicity by the iron-mediated Fenton reaction (Pelizzoni et al. 2011).

In conclusion, the present study demonstrates that intracranially infusion of IONPs has no major adverse impact on neurons in the three brain areas investigated, mPFC, CPu or in the dHip. The consequences observed by the surgical procedure per se or by the injection of an equal amount of the low molecular iron complex FAC appear to be substantially more severe than that of an (additional) IONP injection. This confirms the low toxic potential of DMSA-coated IONPs that has been reported for cultured neural cells (Petters and Dringen 2015) also for the in vivo situation. The Perls’ staining revealed large numbers of iron + microglia after IONP infusion, suggesting that most of the applied IONPs had been internalized by microglial cells. Further studies are now required to analyses whether the application of IONPs to the brain may have consequences on brain functions and animal behavior.

## References

[CR1] Ahmed Z, Shaw G, Sharma VP, Yang C, McGowan E, Dickson DW (2007). Actin-binding proteins coronin-1a and IBA-1 are effective microglial markers for immunohistochemistry. J Histochem Cytochem.

[CR3] Bishop GM, Dang TN, Dringen R, Robinson SR (2011). Accumulation of non-transferrin-bound iron by neurons, astrocytes, and microglia. Neurotox Res.

[CR4] Bishop GM, Robinson SR (2001). Quantitative analysis of cell death and ferritin expression in response to cortical iron: Implications for hypoxia-ischemia and stroke. Brain Res.

[CR5] Boghdadi AG, Teo L, Bourne JA (2020). The neuroprotective role of reactive astrocytes after central nervous system injury. J Neurotrauma.

[CR6] Bulte JWM, Kraitchman DL (2004). Iron oxide MR contrast agents for molecular and cellular imaging. NMR Biomed.

[CR7] Dang TN, Robinson SR, Dringen R, Bishop GM (2011). Uptake, metabolism and toxicity of hemin in cultured neurons. Neurochem Int.

[CR8] Dhakshinamoorthy V, Manickam V, Perumal E (2017). Neurobehavioural toxicity of iron oxide nanoparticles in mice. Neurotox Res.

[CR9] Eng LF (1985). Glial fibrillary acidic protein (GFAP): the major protein of glial intermediate filaments in differentiated astrocytes. J Neuroimmunol.

[CR10] Geppert M, Hohnholt M, Gaetjen L, Grunwald I, Bäumer M, Dringen R (2009). Accumulation of iron oxide nanoparticles by cultured brain astrocytes. J Biomed Nanotechnol.

[CR11] Geppert M, Hohnholt MC, Nürnberger S, Dringen R (2012). Ferritin up-regulation and transient ROS production in cultured brain astrocytes after loading with iron oxide nanoparticles. Acta Biomater.

[CR12] Hayn L, Deppermann L, Koch M (2017). Reduction of the foreign body response and neuroprotection by apyrase and minocycline in chronic cannula implantation in the rat brain. Clin Exp Pharmacol Physiol.

[CR13] Hayn L, Koch M (2015). Suppression of excitotoxicity and foreign body response by memantine in chronic cannula implantation into the rat brain. Brain Res Bull.

[CR14] Ito D, Imai Y, Ohsawa K, Nakajima K, Fukuuchi Y, Kohsaka S (1998). Microglia-specific localisation of a novel calcium binding protein, Iba1. Mol Brain Res.

[CR15] Jain TK, Reddy MK, Morales MA, Leslie-Pelecky DL, Labhasetwar V (2008). Biodistribution, clearance, and biocompatibility of iron oxide magnetic nanoparticles in rats. Mol Pharm.

[CR16] Ji C, Kosman DJ (2015). Molecular mechanisms of non-transferrin-bound and transferring-bound iron uptake in primary hippocampal neurons. J Neurochem.

[CR17] Kawabori M, Kacimi R, Kauppinen T, Calosing C, Kim JY, Hsieh CL, Nakamura MC, Yenari MA (2015). Triggering receptor expressed on myeloid cells 2 (TREM2) deficiency attenuates phagocytic activities of microglia and exacerbates ischemic damage in experimental stroke. J Neurosci.

[CR18] Kwon JT, Hwang SK, Jin H, Kim DS, Minai-Tehrani A, Yoon HJ, Choi M, Yoon TJ, Han DY, Kang YW, Il YB, Lee JK, Cho MH (2008). Body distribution of inhaled fluorescent magnetic nanoparticles in the mice. J Occup Health.

[CR19] Laurent S, Forge D, Port M, Roch A, Robic C, Vander Elst L, Muller RN (2008). Magnetic iron oxide nanoparticles: Synthesis, stabilization, vectorization, physicochemical characterizations and biological applications. Chem Rev.

[CR20] Lorkowski ME, Atukorale PU, Ghaghada KB, Karathanasis E (2021). Stimuli-responsive iron oxide nanotheranostics: a versatile and powerful approach for cancer therapy. Adv Healthc Mater.

[CR21] Luther EM, Petters C, Bulcke F, Kaltz A, Thiel K, Bickmeyer U, Dringen R (2013). Endocytotic uptake of iron oxide nanoparticles by cultured brain microglial cells. Acta Biomater.

[CR22] Mahmoud S, Gharagozloo M, Simard C, Gris D (2019). Astrocytes maintain glutamate homeostasis in the cns by controlling the balance between glutamate uptake and release. Cells.

[CR23] Mai T, Hilt JZ (2019). Functionalization of iron oxide nanoparticles with small molecules and the impact on reactive oxygen species generation for potential cancer therapy. Colloids Surf A Physicochem Eng Asp.

[CR24] Maier-Hauff K, Ulrich F, Nestler D, Niehoff H, Wust P, Thiesen B, Orawa H, Budach V, Jordan A (2011). Efficacy and safety of intratumoral thermotherapy using magnetic iron-oxide nanoparticles combined with external beam radiotherapy on patients with recurrent glioblastoma multiforme. J Neurooncol.

[CR25] Marekova D, Turnovcova K, Sursal TH, Gandhi CD, Jendelova P, Jhanwar-Uniyal M (2020) Potential for treatment of glioblastoma: new aspects of superparamagnetic iron oxide nanoparticles. Anticancer Res 40:5989–5994. 10.21873/anticanres.14619 10.21873/anticanres.14619 33109536

[CR26] Moos T, Møllgård K (1993). A sensitive post-DAB enhancement technique for demonstration of iron in the central nervous system. Histochemistry.

[CR27] Morizawa YM, Hirayama Y, Ohno N, Shibata S, Shigetomi E, Sui Y, Nabekura J, Sato K, Okajima F, Takebayashi H, Okano H, Koizumi S (2017). Reactive astrocytes function as phagocytes after brain ischemia via ABCA1-mediated pathway. Nat Commun.

[CR28] Muldoon LL, Nilaver G, Kroll RA, Pagel MA, Breakefield XO, Chiocca EA, Davidson BL, Weissleder R, Neuwelt EA (1995). Comparison of intracerebral inoculation and osmotic blood-brain barrier disruption for delivery of adenovirus, herpesvirus, and iron oxide particles to normal rat brain. Am J Pathol.

[CR29] Mykhaylyk O, Cherchenko A, Ilkin A, Dudchenko N, Ruditsa V, Novoseletz M, Zozulya Y (2001). Glial brain tumor targeting of magnetite nanoparticles in rats. J Magn Magn Mater.

[CR30] Nel AE, Mädler L, Velegol D, Xia T, Hoek EMV, Somasundaran P, Klaessig F, Castranova V, Thompson M (2009). Understanding biophysicochemical interactions at the nano–bio interface. Nat Mater.

[CR31] Paxinos G, Watson C (1998). The rat brain in stereotaxic coordinates.

[CR32] Pelizzoni I, Macco R, Morini MF, Zacchetti D, Grohovaz F, Codazzi F (2011). Iron handling in hippocampal neurons: Activity-dependent iron entry and mitochondria-mediated neurotoxicity. Aging Cell.

[CR33] Petters C, Dringen R (2014). Comparison of primary and secondary rat astrocyte cultures regarding glucose and glutathione metabolism and the accumulation of iron oxide nanoparticles. Neurochem Res.

[CR34] Petters C, Dringen R (2015). Accumulation of iron oxide nanoparticles by cultured primary neurons. Neurochem Int.

[CR35] Petters C, Irrsack E, Koch M, Dringen R (2014). Uptake and metabolism of iron oxide nanoparticles in brain cells. Neurochem Res.

[CR36] Petters C, Thiel K, Dringen R (2016). Lysosomal iron liberation is responsible for the vulnerability of brain microglial cells to iron oxide nanoparticles: Comparison with neurons and astrocytes. Nanotoxicology.

[CR37] Pickard MR, Chari DM (2010). Robust uptake of magnetic nanoparticles (MNPs) by central nervous system (CNS) microglia: Implications for particle uptake in mixed neural cell populations. Int J Mol Sci.

[CR38] Pinkernelle J, Calatayud P, Goya GF, Fansa H, Keilhoff G (2012). Magnetic nanoparticles in primary neural cell cultures are mainly taken up by microglia. BMC Neurosci.

[CR39] Potter KA, Buck AC, Self WK, Capadona JR (2012). Stab injury and device implantation within the brain results in inversely multiphasic neuroinflammatory and neurodegenerative responses. J Neural Eng.

[CR40] Rastedt W, Thiel K, Dringen R (2017). Uptake of fluorescent iron oxide nanoparticles in C6 glioma cells. Biomed Phys Eng Express.

[CR41] Salehipour M, Rezaei S, Mosafer J, Pakdin-Parizi Z, Motaharian A, Mogharabi-Manzari M (2021). Recent advances in polymer-coated iron oxide nanoparticles as magnetic resonance imaging contrast agents. J Nanoparticle Res.

[CR42] Schindelin J, Arganda-Carreras I, Frise E, Kaynig V, Longair M, Pietzsch T, Preibisch S, Rueden C, Saalfeld S, Schmid B, Tinevez JY, White DJ, Hartenstein V, Eliceiri K, Tomancak P, Cardona A (2012). Fiji: An open-source platform for biological-image analysis. Nat Methods.

[CR43] Schmued LC, Stowers CC, Scallet AC, Xu L (2005). Fluoro-Jade C results in ultra high resolution and contrast labeling of degenerating neurons. Brain Res.

[CR44] Sofroniew MV, Vinters HV (2010). Astrocytes: Biology and pathology. Acta Neuropathol.

[CR45] Urrutia P, Aguirre P, Esparza A, Tapia V, Mena NP, Arredondo M, González-Billault C, Núñez MT (2013). Inflammation alters the expression of DMT1, FPN1 and hepcidin, and it causes iron accumulation in central nervous system cells. J Neurochem.

[CR46] Vakili-Ghartavol R, Momtazi-Borojeni AA, Vakili-Ghartavol Z, Aiyelabegan HT, Jaafari MR, Rezayat SM, Arbabi Bidgoli S (2020). Toxicity assessment of superparamagnetic iron oxide nanoparticles in different tissues. Artif Cells, Nanomedicine Biotechnol.

[CR47] Voinov MA, Pagán JOS, Morrison E, Smirnova TI, Smirnov AI (2011). Surface-mediated production of hydroxyl radicals as a mechanism of iron oxide nanoparticle biotoxicity. J Am Chem Soc.

[CR48] Wang B, Feng W, Zhu M, Wang Y, Wang M, Gu Y, Ouyang H, Wang H, Li M, Zhao Y, Chai Z, Wang H (2009). Neurotoxicity of low-dose repeatedly intranasal instillation of nano- and submicron-sized ferric oxide particles in mice. J Nanoparticle Res.

[CR49] Wang B, Feng WY, Wang M, Shi JW, Zhang F, Ouyang H, Zhao YL, Chai ZF, Huang YY, Xie YN, Wang HF, Wang J (2007). Transport of intranasally instilled fine Fe2O3particles into the brain: Micro-distribution, chemical states, and histopathological observation. Biol Trace Elem Res.

[CR50] Wang FH, Kim DK, Yoshitake T, Johansson SM, Bjelke B, Muhammed M, Kehr J (2011). Diffusion and clearance of superparamagnetic iron oxide nanoparticles infused into the rat striatum studied by MRI and histochemical techniques. Nanotechnology.

[CR51] Wang J, Chen Y, Chen B, Ding J, Xia G, Gao C, Cheng J, Jin N, Zhou Y, Li X, Tang M, Wang XM (2010). Pharmacokinetic parameters and tissue distribution of magnetic Fe(3)O(4) nanoparticles in mice. Int J Nanomedicine.

[CR52] Wang S, Zhang B, Su L, Nie W, Han D, Han G, Zhang H, Chong C, Tan J (2019). Subcellular distributions of iron oxide nanoparticles in rat brains affected by different surface modifications. J Biomed Mater Res A.

[CR53] Wang Y, Wang B, Zhu MT, Li M, Wang HJ, Wang M, Ouyang H, Chai ZF, Feng WY, Zhao YL (2011). Microglial activation, recruitment and phagocytosis as linked phenomena in ferric oxide nanoparticle exposure. Toxicol Lett.

[CR54] White EE, Pai A, Weng Y, Suresh AK, Van Haute D, Pailevanian T, Alizadeh D, Hajimiri A, Badie B, Berlin JM (2015). Functionalized iron oxide nanoparticles for controlling the movement of immune cells. Nanoscale.

[CR55] Willmann W, Dringen R (2018). Monitoring of the cytoskeleton-dependent intracellular trafficking of fluorescent iron oxide nanoparticles by nanoparticle pulse-chase experiments in C6 glioma cells. Neurochem Res.

[CR56] Wolf HK, Buslei R, Schmidt-Kastner R, Schmidt-Kastner PK, Pietsch T, Wiestler OD, Blümcke I (1996). NeuN: A useful neuronal marker for diagnostic histopathology. J Histochem Cytochem.

[CR57] Wu J, Ding T, Sun J (2013). Neurotoxic potential of iron oxide nanoparticles in the rat brain striatum and hippocampus. Neurotoxicology.

[CR58] Xie W, Guo Z, Gao F, Gao Q, Wang D, Liaw BS, Cai Q, Sun X, Wang X, Zhao L (2018). Shape-, size-and structure-controlled synthesis and biocompatibility of iron oxide nanoparticles for magnetic theranostics. Theranostics.

[CR59] Yarjanli Z, Ghaedi K, Esmaeili A, Rahgozar S, Zarrabi A (2017). Iron oxide nanoparticles may damage to the neural tissue through iron accumulation, oxidative stress, and protein aggregation. BMC Neurosci.

[CR60] Yasuda Y, Shinagawa R, Yamada M, Mori T, Tateishi N, Fujita S (2007). Long-lasting reactive changes observed in microglia in the striatal and substantia nigral of mice after 1-methyl-4-phenyl-1,2,3,6-tetrahydropyridine. Brain Res.

[CR61] Zhou YF, Zhang C, Yang G, Qian ZM, Zhang MW, Ma J, Zhang FL, Ke Y (2017). Hepcidin protects neuron from hemin-mediated injury by reducing iron. Front Physiol.

